# Rapid Estimation of Water Stress in Choy Sum (*Brassica chinensis* var. *parachinensis*) Using Integrative Approach

**DOI:** 10.3390/s22051695

**Published:** 2022-02-22

**Authors:** Alaa AL Aasmi, Kelvin Edom Alordzinu, Jiuhao Li, Yubin Lan, Sadick Amoakohene Appiah, Songyang Qiao

**Affiliations:** 1 College of Water Conservancy and Civil Engineering, South China Agriculture University, Wushan Road No. 483, Guangzhou 510642, China; alaaasmi83@gmail.com (A.A.A.); kelvinedomalordzinu@gmail.com (K.E.A.); 20191000028@stu.scau.edu.cn (S.A.A.); qsy1999@stu.scau.edu.cn (S.Q.); 2 National Center for International Collaboration Research on Precision Agricultural Aviation Pesticides Spraying Technology (NPAAC), College of Engineering, South China Agriculture University, Wushan Road No. 483, Guangzhou 510642, China; ylan@scau.edu.cn

**Keywords:** crop water stress index, Vegetation indices, choy sum plants, water stress indicators, Photochemical Reflectance

## Abstract

To optimize crop water consumption and adopt water-saving measures such as precision irrigation, early identification of plant water status is critical. This study explores the effectiveness of estimating water stress in choy sum (*Brassica chinensis* var. *parachinensis*) grown in pots in greenhouse conditions using Crop Water Stress Index (CWSI) and crop vegetation indicators to improve irrigation water management. Data on CWSI and Spectral reflectance were collected from choy sum plants growing in sandy loam soil with four different soil field capacities (FC): 90–100% FC as no water stress (NWS); 80–90% FC for light water stress (LWS); 70–80% FC for moderate water stress (MWS); and 60–70% FC for severe water stress (SWS). With four treatments and three replications, the experiment was set up as a completely randomized design (CRD). Throughout the growing season, plant water stress tracers such as leaf area index (LAI), canopy temperature (Tc), leaf relative water content (LRWC), leaf chlorophyll content, and yield were measured. Furthermore, CWSI estimated from the Workswell Wiris Agro R Infrared Camera (CWSI_W_) and spectral data acquisition from the Analytical Spectral Device on choy sum plants were studied at each growth stage. NDVI, Photochemical Reflectance Index positioned at 570 nm (PRI570), normalized PRI (PRInorm), Water Index (WI), and NDWI were the Vegetation indices (VIs) used in this study. At each growth stage, the connections between these CWSI_W_, VIs, and water stress indicators were statistically analyzed with R^2^ greater than 0.5. The results revealed that all VIs were valuable guides for diagnosing water stress in choy sum. CWSI_W_ obtained from this study showed that Workswell Wiris Agro R Infrared Camera mounted on proximal remote sensing platform for assessing water stress in choy sum plant was rapid, non-destructive, and user friendly. Therefore, integrating CWSI_W_ and VIs approach gives a more rapid and accurate approach for detecting water stress in choy sum grown under greenhouse conditions to optimize yield by reducing water loss and enhancing food security and sustainability.

## 1. Introduction

Optimization of crop water use through early and accurate detection of water stress in crop production is very vital in sustainable agriculture. Owing to the overwhelming effect of global warming and scarcity of water resources on agricultural production, there is the need for more simple, accurate, non-destructive, and user-friendly methods of estimating plant water stress [1]. Most researchers have used various methods to evaluate crop water status, which includes the use of soil moisture monitoring tools. This approach is laborious, time-consuming, and necessitates the use of a huge number of soil moisture sensing tools to explain the longitudinal variation of the soil properties [2,3,4]. Due to the increasing human population and high food demand, there is the need to unswervingly monitor crop water conditions by observing the biophysical and morphological responses of plants to water stress [5]. Plants that are stressed by water absorb more incident radiation energy than is obligatory for their physiological processes, resulting in a surplus of photosynthetic demand [6,7]. To minimize damage to the photosynthetic pigment, the plants waste this surplus energy as chlorophyll fluorescence and heat resulting in growth and yield reduction [8,9]. Correspondingly, crops close their stomata to preserve water during biotic and abiotic stress conditions, thereby altering their enzymatic and biochemical pathways. In choy sum, the same as any other leafy vegetable, reduction in crop water denatures the leaf morphology causing a reduction in transpiration and photosynthetic rate, thereby increasing the leaf temperature and reducing crop growth and yield [10,11,12]. During increasing environmental temperatures, plants shut their stomatal pores to improve the water balance and fluctuations in transpiration intensity which generally regulate the degree of heat emitted by plant leaves, leading to leaf temperature variation. In the study of crop physiological features and environmental science, foliage temperature is an important variable [13,14,15]. Crop cover temperature can be employed to determine crop water status since it imitates water and heat exchange amongst plants and the environment [16]. However, using infrared thermometers to obtain leaf temperature and air temperature, for diagnosing the effect of drought in crops, Crop Water Stress Index (CWSI) was calculated and created [17,18]. The use of infrared thermometers to assess leaf temperature is a useful tool for detecting crop water deficiencies. According to Poblete-Echeverría et al. [15], crop leaf water status is directly proportional to leaf temperature, however significant progress has been made in recent times on the utilization of leaf temperature as a very sensitive tool to monitor crop water stress in a more easy, accurate and reliable manner [8,19]. When the soil moisture content is 100% field capacity (FC), efficient transpiration occurs, increasing the cooling impact on leaves and decreasing leaf temperature below the ambient temperature [20] Several leafy vegetables, such as choy sum, require a lot of water and are vulnerable to water stress, which can harm their functional and biochemical growth. CWSI is by far the most commonly used indicator for detecting agricultural water shortages because it is based on leaf temperature. CWSI was created as a normalized index to identify water stress and to offset the impacts of other factors in the environment that alter the stress-plant temperature association [2,21,22]. The CWSI is frequently calculated using empirical methods that relate the canopy temperature (Tc) and air temperature (Ta) differential (Tc-Ta) to a non-water-stressed baseline’s air vapor pressure deficit. However, many researchers have measured different crops including cabbage leaf temperatures under various irrigation regimes to establish a CWSI archetypal that could trail crop water status in real-time [23,24]. To establish CWSI, it is very important to determine the upper and lower limit baseline. Alordzinu et al. [2] used Workswell Wiris Agro R infrared camera thermal image processed in CorePlayer to estimate both upper and lower limit equations in various soil texture types at varying stages of tomato growth. Lately, several studies have also applied high-resolution proximal and remote sensing devices to perceive variances in canopy temperature for measuring the water stress in vegetations [3,5,13,25] since leaf temperature is a direct index of plant transpiration. Numerous narrow-band hyperspectral indices for assessing crop water stress have been investigated recently, this approach utilizes spectral reflectance ranging from the visible light (380 nm) to the infrared (1300 nm) range. The spectrum features of plant leaves are influenced by these spectra’s reflectances, providing cost-effective, accurate, precise, and adequate data on crop stresses. Several spectral Vegetation indices have been studied in the past for monitoring water stress in crops, including the Normalized Difference Vegetation Index (NDVI), Renormalized Difference Vegetation Index (RDVI), Normalized PRI (PRInorm), and Normalized Difference Water Index (NDWI). In recent years, many studies have used soil moisture, leaf relative water content (LRWC), leaf area index (LAI), and leaf chlorophyll content (LCC) to effectively estimate water stress in crops [26]. The principal objective of this research was to (1) evaluate the relation between the CWSI estimated from the Workswell Wiris Agro R Infrared Camera (CWSI_W_) and plant water stress indicators, (2) assess the viability of the CWSI_W_ in forecasting the morphological development and chlorophyll content (SPAD values) of choy sum, (3) analyze and compare different spectral VIs for water stress monitoring in choy sum plants and (4) elucidate the relationship between the CWSI_W_ and Vis. These objectives are targeted at the rapid and accurate estimation of CWSI_W_’s suitability for monitoring choy sum growth and identifying water deficits.

## 2. Materials and Methods

### 2.1. Irrigation Treatments and Study Designs

This study was conducted at the Tea Research Institute of Guangdong Academy of Agricultural Sciences, located in Guangzhou (23°13′ N, 113°81′ E, altitude 11 m), China. The region’s climate is subtropical monsoon. A simple arc, 120 m^2^ greenhouse covered with a diffused polyethylene sheet to allow for uniform light distribution to all areas in the greenhouse was used. The greenhouse floor was covered with white woven polyethylene to control weeds, prevent waterlogging and increase light reflection for maximum photosynthesis. Maximum and minimum temperature, and humidity in the greenhouse were measured daily throughout the cropping season and the mean values at each growth stage were recorded as shown in Table 1. Choy sum (*Brassica chinensis* var. *parachinensis*) seeds were planted at stake on the first week of January, germinated seeds were thinned-out to 4 plants per pot. Each pot was 20 cm deep and 40 cm in diameter. The soil used was sandy loam, and the physicochemical parameters of the soil are listed in Table 2. The pots were randomly placed in the greenhouse and were spaced at a distance of 20 cm × 40 cm. To guarantee that the soil water content was at field capacity, the pots were filled and allowed to drain for 48 h before sowing. The experiment was organized in a randomized complete block design constituting a 4 × 3 factorial experiment with two factors (water regime and growth stage) and three replications. The first factor was the water regime, which included four distinct water-management strategies: (i) wetting to reach the soil moisture content near field capacity 90–100% FC as no water stress (NWS); (ii) wetting to reach the soil moisture content to 80–90% FC as light water stress (LWS); (iii) wetting to reach the soil moisture content to 70–80% FC as moderate water stress (MWS); (iv) wetting to reach the soil moisture content to 60–70% FC as severe water stress (SWS). The second factor was the growth stages (the stage to apply water stress), which were divided to three stages: initial growth stage, vegetative stage, and flowering stage. Accordingly, 36 experimental pots (4 irrigation regimes × 3 growth stages × 3 replicates) were set up under the greenhouse. The variance between the field capacity and the permanent wilting point of the soil was described as the soil available water (AWC), and the soil water content (SWC) in each pot was continuously measured with soil moisture sensors. For each treatment, the upper irrigation barrier was set at 90–100% FC, 80–90% FC, 70–80% FC, and 60–70% FC, while the lower irrigation barrier was set at a 50% AWC. When the SWC in the pots reached the low limit, irrigation commenced and halted when the target soil field capacity was reached. A drip irrigation technology with emitters and a discharge rate of 2 L h^−1^ was used to calibrate the flow rates of high-quality irrigation water in the greenhouse. All treatments received consistent irrigation at the start of transplanting, dependent on a 100% FC replenishment of water in the plant root zone to field capacity for plants to develop throughout their development phase. The actual volumetric soil water content was determined using an HD2 precise moisture measurement TDR instrument by IMKO, Germany, after calibrating the device to set benchmarks before water application at the four irrigation water levels. TDR stayed planted at a soil thickness of 20 cm vertically from the top of the soil surface, which corresponded to the choy sum’s root and pot depths. When the plants grew their third true leaves, water stress treatment was applied. Nutrient-free freshwater was utilized throughout the experiment, because the soil analyses revealed that the soil was fertile enough to support the plant’s growth, no fertilizer was used.

### 2.2. Field Data Collection

#### 2.2.1. Leaf Temperature and Air Temperature Measurement

The temperature of the leaves was checked using a Raytek^®^ quasi-infrared thermometer with an absorption coefficient of 0.95 W/m^2^ having a displaying resolution of 0.2 °C (0.5 °F). The thermometer was set 30 cm above the choy sum leaf, with the optical maser point at a 90-degree angle to the target leaf [3,27]. At each development phase of the choy sum active growing phases, temperature readings were taken on five plants from each treatment. Commencing from the northern part, the temperatures of each leaf in individual treatment were measured around the plant, and the mean temperatures for each leaf were calculated. Ihuoma and Madramootoo [3] recommended taking temperature readings on properly formed leaves at the crop’s apex at 11:00 a.m. and 2:00 p.m. in the greenhouse. In the greenhouse, the temperature and relative humidity were measured using a portable Vip Gadgets Dawn Labs ABS Multifunctional Digital Thermometer Hygrometer (Manufacturer:dawnlabs, Kolkata, India).

#### 2.2.2. Leaf Chlorophyll Content (LCC), Leaf Relative Water Content (LRWC) and Leaf Area Index (LAI) Measurement

The SPAD-502 Plus handheld chlorophyll meter (Konica Minolta Optics, Osaka, Japan) is a diagnostic tool for determining the amount of chlorophyll in a crop’s leaves. SPAD values on the freshest completely formed leaf were taken at every developmental phase. The following equation [21] was used to convert the SPAD values to N-sufficiency Index:

(1) NSI=SPAD(target)/SPAD(reference)

At each stage of plant development, leaf relative water content (LRWC) was determined by selecting immature and properly formed leaves from four plants within every treatment. The fresh leaves selected were placed in a rubber Ziploc bag and stored at 5 °C before being brought to the lab. The fresh (FW) and dry weight (DW) of the leaves were determined. The weight was measured and recorded on an electronic scale. To obtain the turgid weight, samples were immersed in distilled water for 72 h, blotted, and weighed (TW). Finally, the leaf samples were dried in an oven at 72 °C until they reached a consistent dry weight (DW). The following equation [13,28] was used to compute the relative water content of the leaves.

(2) LRWC=(FM−DM)/(TM−DM)×100

where LRWC% is the Leaf Relative Water Content, FM stands for fresh leaf mass, DM stands for dry leaf mass, and TM stands for turgid leaf mass.

Leaf area index (LAI) was calculated according to [29] as

(3) $$\mathrm{SLA}=\frac{\mathrm{Total}\;\mathrm{leaf}\;\mathrm{area}}{\mathrm{Total}\;\mathrm{leaf}\;\mathrm{dry}\;\mathrm{weight}}$$

LAI = W_greenleaves_ × SLA (4)

where W_greenleaves_ is the weight of green leaves (g dry matter m^−2^ total ground area); and SLA is the specific leaf area (m^2^ leaf area g^−1^ dry matter leaf).

#### 2.2.3. Root Morphology Measurements

Roots were extracted by thoroughly cleaning the soil, centrifuging the residual water, measuring root fresh weight (RFW), oven-drying the root subsample at 75 °C for 72 h, and calculating root dry weight (RDW) using the fresh weight and moisture content of the roots, via the method used by [30]. After root fresh weight (RFW) was measured, total root length (RL) was estimated by the line intersection method described by [31]. Roots volume was measured by the water-displacement method [32]. Root length density (RLD) (mm/cm^3^), root weight density (RWD) (g/cm^3^), specific root length (SRL) (m/g), root total surface area (TSA) (m^2^), and root diameter (RD) (mm) were calculated by using the following equations:

(5) RLD= RL /V

(6) RWD=RDW/V

(7) SRL=RL/RDW

(8) $$\mathrm{RD}={\left(\mathrm{RDW}/\mathrm{RL}\times \pi \right)}^{0.5}$$

where, RLD root length density, RL root length (cm), V soil volume (cm^3^) and RDW root dry weight (g).

Supposing that fresh roots are cylindrical with 1.0 g/cm^3^ density [33], the mean radius of root (r_0_) and total surface area TSA was determined from the equations:

(9) $$\mathrm{TSA}=2\times \pi \times r_{0}\times \mathrm{RL}$$

(10) $$r_{0}={\left(\mathrm{RFW}/\mathrm{RL}\times \pi \right)}^{0.5}$$

where RFW root fresh weight (g).

#### 2.2.4. Image Acquisition and Estimation of the CWSI_W_

WWARIC contains four-color maps with complete radiometric (temperature) information for determining CWSI_W_. The digital CWSI_W_ image, CWSIw video, and Visible images are all acquired in real-time during the Intermittent image acquisition. Full-Screen RGB with subdivision has a Dual-screen Micro HDMI video output of 1280 × 720 pixels (720 p), and a functionality ratio of 16:9 with HDMI and SDK video output software for computers. The innovative Workswell CorePlayer for offline CWSI_W_ images analysis includes an input power source that ranges from 9 to 36 DCV, a co-axial of 2 × 6.4 mm, and a 12 watt outmost cover GND Power degeneracy. The WWARIC is 430 g in weight and measures 83 mm in length, 85 mm in width, and 68 mm in height. This camera can be used with a tripod stand, UAV, and satellite system installed on a 21/4–20 UNC support system. The camera case is made up of a rigid aluminum plate to withstand external heat and protect the internal system from damage. It can operate in environmental temperature (lower temperature of −10 °C and highest temperatures of 50 °C and can be well stored at minimum temperature of 30 °C and maximum temperature of 60 °C. The Workswell WIRIS Agro R is a thermal infrared camera (WWARIC) that is used in precision agriculture to map crop water status. The WWARIC was placed at a height of 4.5 m above the greenhouse plant and several images were taken from different perspectives. To evaluate water stress in choy sum, images collected with WWARIC were processed in CorePlayer version 3.18 in three color modes: empirical mode, theoretical model, and differential mode. This data can subsequently be utilized to create yield maps, improve irrigation, and control water management solutions because the technology makes determining the crop water stress index value in a given plant stand much easier because it allows for the region of interest estimation, which provides the CWSI_W_ values automatically within a given region of interest (ROI).

#### 2.2.5. Acquisition and Processing of Spectral Data

The reflectance of choy sum leaves was obtained utilizing an Analytical Spectral Device (ASD) FieldSpec 4 (FS4). The ASD Field Spec 4 Hi-Res spectroradiometer provides superior spectral performance across the full range solar irradiance spectrum (350–2500 nm). The enhanced spectral resolution in the VNIR ranges from 350 nm to 1000 nm whilst the SWIR ranges from 1001 nm to 2500 nm. The instrument was calibrated before measurement on each treatment was taken.

To reduce integration time, the probe was rotated 45 degrees around a white standard [34] (typically set at 50 ms), capturing dark current and then obtaining target reflectance at a distance of roughly 60 mm. To ensure maximum sun intensity, all canopy spectral measurements were obtained between 11 a.m. and 1 p.m. under clear skies. Narrow-band spectral Vegetation indices (SVIs) were chosen for study because they are capable of assessing the vegetation parameters relating to the health, morphological, and biochemical characteristics of crops. Crop photosynthetic activity and canopy structural modifications can be reliably compared geographically and temporally using these spectral modifications of two or more bands. As a result, it is a very valuable tool for evaluating environmental issues or global warming. NDVI, RDVI, CLgreen NDWI, and PRInorm were the VIs employed in this work. These data are shown in Table 3.

### 2.3. Statistical Analysis

Choy sum leaf temperature (LT °C), RLWC%, LCC (mg/g), and LAI (m^2^/m^2^), as well as spectrum vegetative indices (VIs) (NDVI, RDVI, CL_green_, NDWI, and PRI_norm_) among treatments and blocks, as well as CWSI_W_ at each growth stage, were interactively utilized to assess water stress in choy sum plants. The influence of soil FC% of the soil properties on choy sum growth was a continuous scale variable with a normal distribution, a two-tailed test of significance was performed using Pearson correlation coefficient. The correlation was assumed to have a value between −1 and +1, which elucidates the fluctuation in water stress data. A negative number represents a perfect negative correlation, whereas a positive number represents a strong positive correlation. Fisher Least Significant Difference was utilized to differentiate significant differences across treatments at the 0.001, 0.01, and 0.05 significant levels. The association between spectral reflectance factors and water stress indicators was investigated using a bivariate analysis with a two-tailed test in IBM SPSS statistic 21.

## 3. Result and Discussion

**Figure 1:**
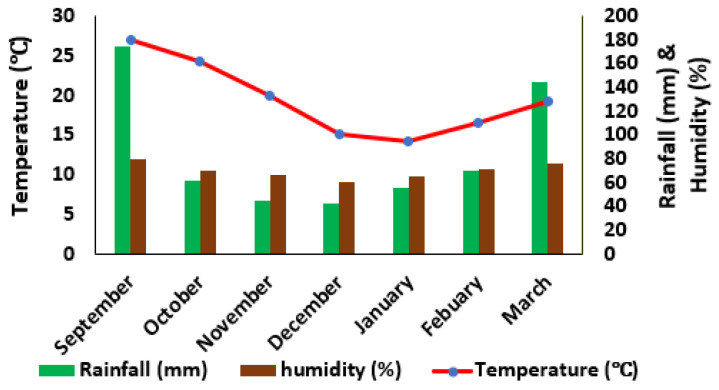
Average temperature (°C), rainfall (mm) and relative humidity (%) data recorded during the study period.

**Figure 2:**
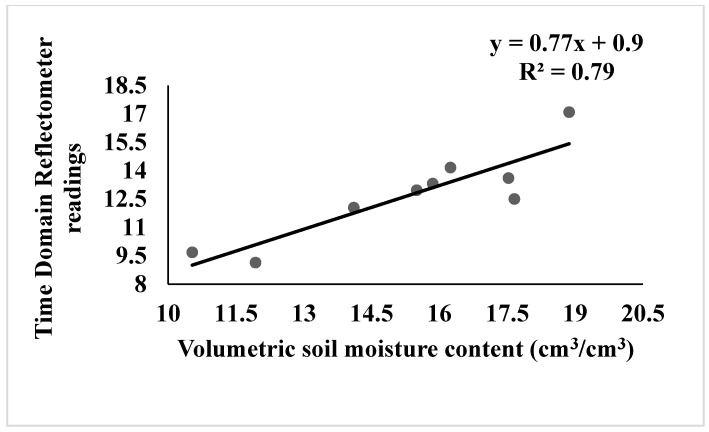
Correlation between TDR and VWC readings and soil Volumetric Water Content.

Figure 1 depicts the monthly mean temperature, relative humidity, and rainfall during the plant growing season. Figure 2 depicts the correlation between Volumetric water content (VWC) and Time Domain Reflectometer (TDR) revealing a strong correlation at R^2^ = 0.79. The coefficient of correlation between volumetric water content and TDR was observed to be improved in this study, proving that TDR equipment may be used solely to estimate soil water content in this type of soil with ease. The association between the VWC and TDR measurements was significant (*p* ≤ 0.05) after the statistical analysis. The findings of this study were comparable to those of [36,37] who reported R^2^ values ranging from 0.65 to 0.99 as the correlation coefficient between VWC and TDR.

### 3.1. Variation in Volumetric Soil Water Content

**Figure 3:**
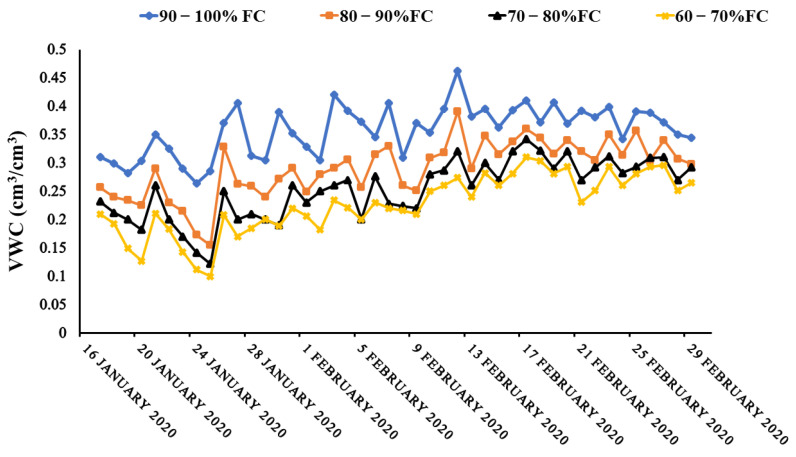
Variation in volumetric water content during the growth season of choy sum.

The amount of water in the soil was affected by irrigation regimens. The fluctuation in volumetric water content during the planting season is depicted in Figure 3. There was a reduction in water application with reducing in soil field capacity from 90–100% FC to 60–70% FC, resulting in the average highest and lowest soil water content values, for 90–100% FC, 80–90% FC, 70–80% FC, and 60–70% FC, respectively, over the growing season. The soil moisture content at 90–100% FC, 80–90% FC, 70–80% FC, and 60–70% FC treatments, however, varied significantly, especially when daily temperatures increase at which the crops needed more water for biophysical and physiological growth and development. These results conform to the findings of [14,38,39,40,41].

### 3.2. Features of Spectral Reflectance in Cropping

**Figure 4:**
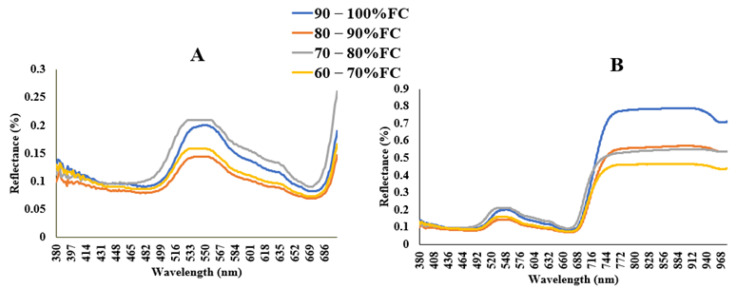
(**A**) Examples of leaf spectral signatures for various treatments in the visible light region (380 nm to 700 nm), (**B**) leaf spectral reflectance in the visible region to the near infrared region (380 nm to 980 nm).

Figure 4A,B depicts the average spectral reflectance of choy sum leaves grown with different field capacities. The leaf reflectance varied depending on the irrigation treatments, demonstrating that water stress influenced leaf spectral characteristics for peaks near 545 nm, and 740 nm, for visible light and NIR regions, respectively. In this study, the spectral values from water-stressed and non-water-stressed plants were obtained from the visible spectrum region (380 nm to 648 nm) and NIR spectrum (701 nm to 980 nm) to estimate crop water status in choy sum. The reflectance wavelengths observed from choy sum leaves were similar to that of [3,13,42] who used narrow-band hyperspectral reflectance to estimate crop water status using VIs.

### 3.3. Correlation between Vegetative Indices and Leaf Temperature °C, Leaf Area Index, Relative Leaf Water Content, and Leaf Chlorophyll Content

**Figure 5:**
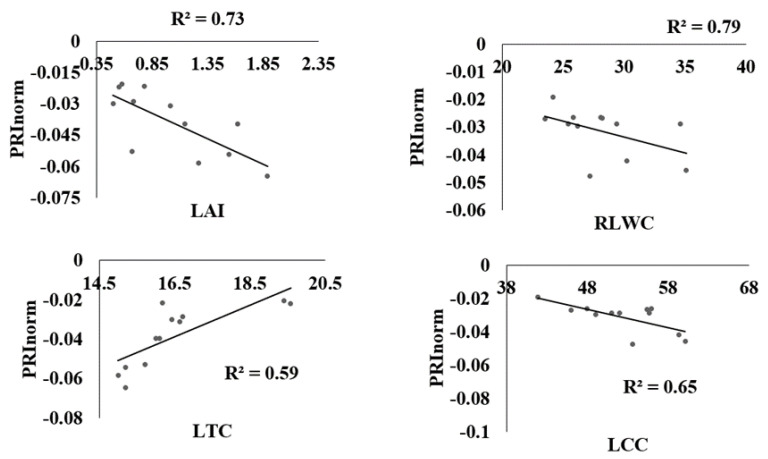
Relationship between PRInorm, leaf temperature, Leaf Area Index, Leaf Chlorophyll Content, and Relative Leaf Water Content found from the various treatments.

**Figure 6:**
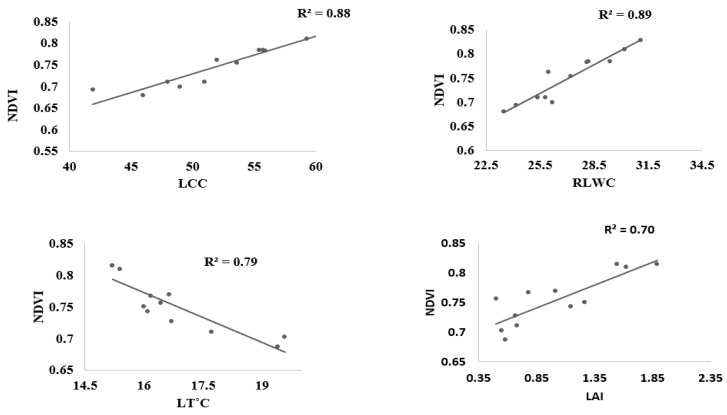
Association between NDVI, leaf temperature, Leaf Area Index, Leaf Chlorophyll Content, and Relative Leaf Water Content found from the numerous treatments.

**Figure 7:**
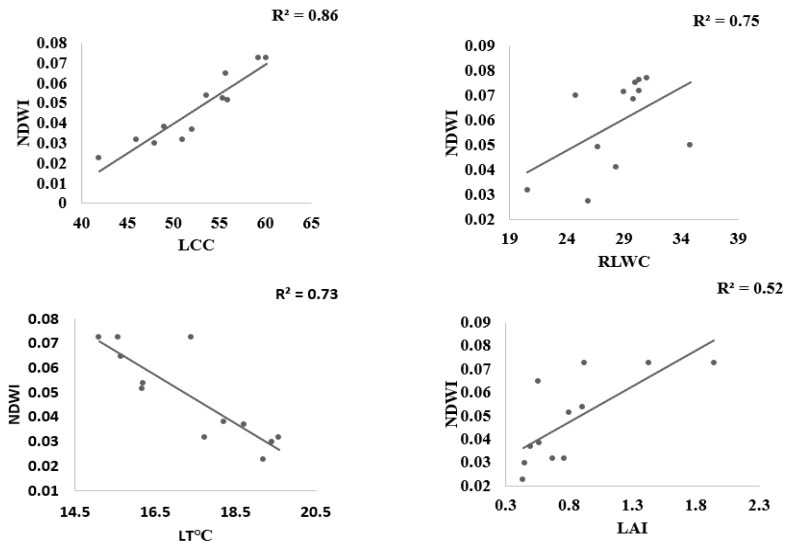
Association between NDWI, leaf temperature, Leaf Area Index, Leaf Chlorophyll Content, and Relative Leaf Water Content found from the numerous treatments.

**Figure 8:**
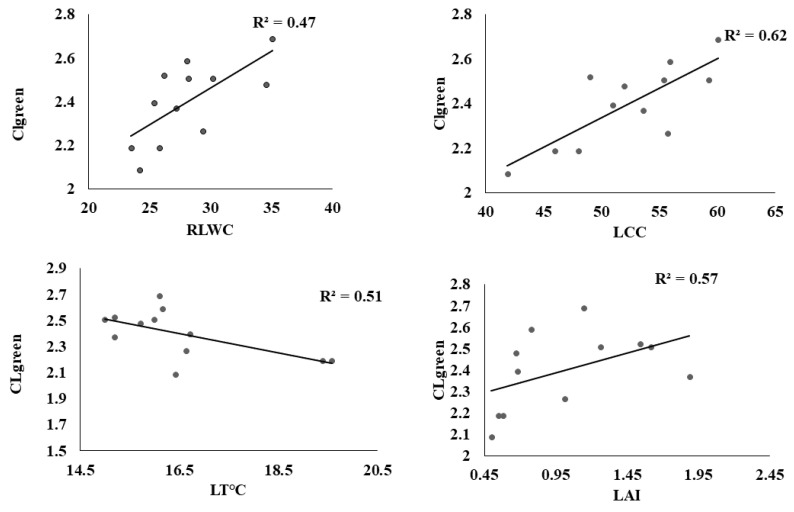
Association amongst CLgreen, leaf temperature, Leaf Area Index, Leaf Chlorophyll Content, and Relative Leaf Water Content obtained from the various treatments.

The coefficient of determination R^2^ of the linear relations between LT °C, LAI, LRWC, LCC, and spectral reflectance indices computed from the ASD hyperspectral spectrometer has been utilized by many researchers for stress estimation, but this has been less applicable to estimating water stress in leafy vegetables, especially in choy sum. Figure 5 shows that PRInorm was strongly correlated with LT °C, LAI, RLWC, and LCC at R^2^ = 0.74, 0.75, 0.79, and 0.65 respectively at *p* ≤ 0.05. similarly, NDVI also presented a significant association with all water stress indicators at R^2^ above 0.70 as shown in Figure 6. NDWI was strongly correlated with LT °C, LAI, RLWC, and LCC at R^2^ 0.73, 0.69, 0.75, and 0.86, respectively, at *p* ≤ 0.05 as shown in Figure 7. Correspondingly, CL_green_ also revealed a strong correlation with LCC, LT °C, LAI, LRWC at R^2^ above 0.5 at *p* ≤ 0.05) as shown in Figure 8. The results reveal that water stress had a substantial impact on all of the Vegetation indices employed in this study, indicating the possibility of predicting water stress. However, subsequent empirical research reveals that PRInorm, NDVI, and NDWI did best in identifying the impacts of water stress in choy sum leaves, but CLgreen performed better.

Within the visible spectrum, the choy sum leaves stressed at 70–80% FC had very high reflectance values compared to the plant leaves stressed at 80–90% FC, 60–70% FC and even the no water stress plants. In general, plant pigments absorb more photons in the visible light spectrum but reflect the vast majority of them in the near-infrared (NIR) spectrum. Plant stress affects this spectral reflectance pattern, resulting in increased reflectance in the visible band and decreased reflectance in the NIR band due to reduced photosynthetic pigment efficiency. Researchers have computed structural indices (such as NDVI) based on this notion [3,38]. Water stress had the most pronounced impacts, with the highest reflectance values in the visible region, implying that stress influenced the concentration of leaf photosynthetic pigment. The non-water-stressed groups had the lowest reflectance values in the 690 nm band, implying that stress decreased leaf chlorophyll content, lowering chlorophyll absorption at the trough. The findings demonstrate that changes in leaf spectral reflectance are related to differences in water stress, highlighting the utility of this method for evaluating water stress in leafy crops and optimizing productivity. According to [43], water-stressed crops constrict their stomata to limit the amount of water lost during transpiration. However, [21] found that when plants are exposed to high water stress, their leaves become dehydrated and have a weaker cell wall, lowering their cell turgor pressure. The decrease in RLWC seen in water-stressed treatments when compared to non-stressed treatments was linked to a decrease in cell wall turgor pressure. Plants dissipate surplus energy as heat by the changing light energy from sunlight absorbed in the xanthophyll pigment and chlorophyll fluorescence, according to El-Shirbeny and Abutaleb [14], Gamon et al. [44], Rossini et al. [45], Suárez et al. [7], and Zarco-Tejada et al. [46]. This study discovered a decrease in RLWC, LAI, and LCC in water stress treatments at 60–70% FC compared to non-water stress treatments at 90–100% FC, implying that water stress treatments affect leaf chlorophyll content and increase leaf temperature, denaturing the plants’ physiological and enzymatic processes. Other major research findings [2,3,13,47] are similar to the findings of this study.

### 3.4. Correlation between Water Stress Indicators and CWSI_W_

**Figure 9:**
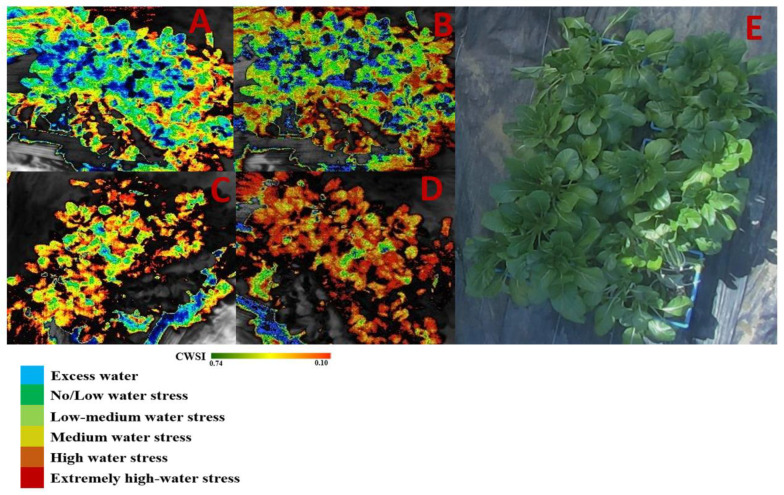
Spatial and temporal changes of CWSI_W_. Spatial distribution of CWSI_W_ obtained from Workswell Wiris Agro R Infrared Camera at (**A**), CWSI_W_ 90–100% FC (**B**), CWSI_W_ 80–90% FC (**C**) CWSI_W_ 70–80% FC, (**D**) CWSI_W_ 60–70% FC, and (**E**) is the RGB digital image of choy sum.

**Figure 10:**
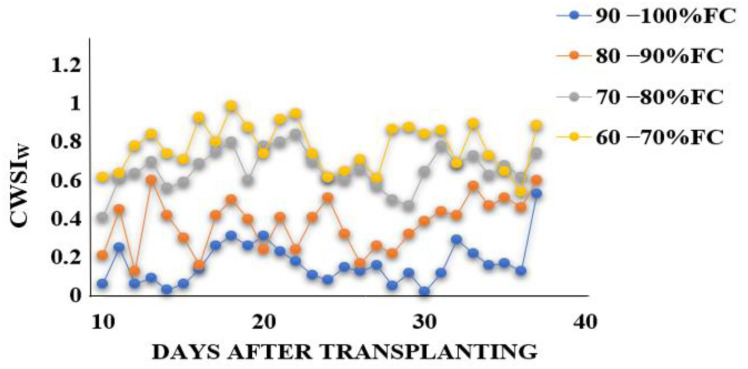
CWSI_W_ values change at different soil field capacities during the growing season.

**Figure 11:**
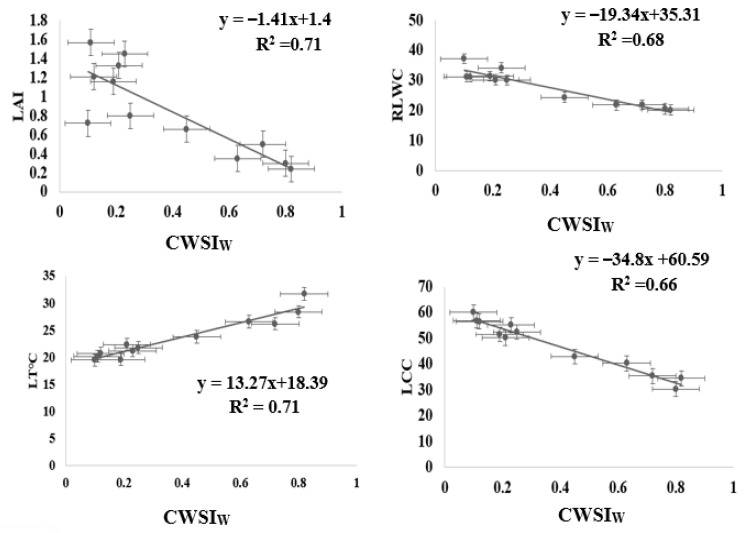
Relationship between CWSI_W_, leaf temperature, Leaf Area Index, Leaf Chlorophyll Content, and Relative Leaf Water Content obtained from the various treatments.

Figure 9 illustrates the spatial and temporal changes in CWSI_W_ recorded from the Workswell Wiris Agro R infrared camera and evaluated with the CorePlayer version 1.3.8. Figure 10 also revealed the CWSI_W_ values changes at different soil field capacities during the growing season. This experiment found a substantial association between CWSI_W_ and water stress indicators on choy sum leaves, with R^2^ values above 0.65 at each growth stage. This is linked to the stages of leaf growth, flower development stage, and chlorophyll pigment formation, during which the plant consumes a lot of water for biochemical and enzymatic processes. However, Figure 11 demonstrated a significant association between CWSI_W_ and LT °C, LRWC, LAI, and LCC at R^2^ = 0.71, 0.68, 0.71, and 0.65 for all color modes employed at each growth stage of choy sum development. The empirical and theoretical modes of the Workswell Wiris Agro R Infrared Camera, according to Alordzinu et al., (2021) [2], are capable of estimating water stress at a height of 4–10 m, and this was related to vegetable plants. When there is little vegetation on the soil surface, however, the majority of remote sensing reports display a greater percentage of varied pixels [2,15,21]. Crop traits, not soil conditions, have the greatest influence on CWSI_W_ [2,21,47,48] These findings show that utilizing the Workswell Wiris Agro R Infrared Camera to approximate crop water stress index in choy sum is both accurate and quick. This study’s findings are equivalent to those of [2,49].

### 3.5. Correlation between CWSI_W_ and VIs

Table 4 demonstrates that CWSI_W_ from WWARIC was significantly correlated with NDVI, PRInorm, NDWI, and CL_green_ with R2 greater than 0.60 and *p* ≤ 0.001. The VIs utilized in this study were extremely useful for evaluating crop water status and validating spectral reflectance indices for detecting water stress in the plant canopy. According to these findings, the best-linked variables with CWSI_W_ were PRInorm, NDVI NDWI, and CL_green_. Greenness vegetative index, water index, and photochemical index have a high correlation with CWSI_W_ in forecasting plant water stress, according to, Idso [50] and Ru et al. [21]. The fluctuation of the quantity of water in the soil is a feature of the amount of water in the plant leaves that connects all of the VIs with CWSI_W_. According to Alordzinu et al. [2], Poblete-Echeverra et al. [15] and Tanriverdi et al. [37] CWSI_W_ for monitoring agricultural water status is especially fast and sensitive to water stress estimation.

### 3.6. Effect of Water Stress on Root Morphological Traits of Choy Sum

The root morphological properties were measured at the end of the planting season and mean values were recorded for every treatment. The roots from the 90–100% FC were greater than those 80–90% FC, 70–80% FC, and 60–70% FC. The greatest ARL was 7870 ± 96.8 cm at *p* ≤ 0.05 in the 90–100% FC, whereas the 60–70% FC recorded the least ARL at 3921 ± 32.1 cm *p* ≤ 0.05. ARL for choy sum in each treatment was significantly different from each other at *p* ≤ 0.05. The roots of choy sum from the 90–100% FC exhibited an increase in RLD at 4.75 ± 0.97 and a decrease in 1.38 ± 0.72 for 60–70% FC at *p* ≤ 0.05 with no significant difference between 70–80% FC and 80–90% FC, these results are similar to the results TSA, RFW, and SRL as shown in Table 5. However, it was evident that root-form 90–100% FC showed an increase in RDW.

The morphological characteristics of choy sum roots as quantified in this study revealed that even though reduced soil water content affects plant roots, maintaining the water content in the soil throughout the growing season improves yield and plants adaptability. It is well known that root systems can change their morphological structure in response to the soil environmental conditions such as temperature and soil water content [51]. Root development in choy sum was affected by the soil moisture content in this study. Root development was better under the 90–100% FC treatment than in 60–70% FC for choy sum growth but improved in the 70–80% FC and 80–90% FC treatment. Specifically, the roots were longer, deeper, more numerous, and covered a wider surface area in the 90–100% FC treatment than in the other treatment with reduced soil field capacity. According to Chun et al. [52], when the soil water content decreases, plant roots exhibit significantly slow and poor development. However, many studies have reported that crop roots tend to grow deeper into the soil when the immediate soil the root occupies is deficient in water [39,53,54,55,56,57,58] with one study reporting that root growth of plants in water stress conditions reduced root development and may cause root damage. Chung et al. and Zhang et al. [51,58] reported that the drought level and root growth were inversely related if the drought occurred from the onset of planting or early growth stages. Results from this study inveterate that severe water stress limits root growth throughout the choy sum growth especially during the early and flower development stages. The poor root development at the seedling stage decreased choy sum productivity; this result is consistent with the findings of [59,60,61].

## 4. Conclusions

The application of CWSI_W_, Vegetation indices, water stress indicators, and root morphological features to determine the impacts of water in choy sum plants was investigated in this study. The VIs utilized in this study were all sensitive indices for diagnosing the impacts of water stress on choy sum, according to a comparison of numerous VIs indices and their relationship with stress parameters. The best correlations with water stress indicators were CWSI_W_, NDWI NDVI, PRInorm, and CL_green_. This study confirmed that integrating the use of Workswell Wiris Agro R infrared camera in estimating water stress with Vegetation indices obtained from ASD hyperspectral reflectance device to simultaneously estimate water status in choy sum is rapid, non-destructive, and not laborious to enhance precision irrigation and water management. However, root morphological traits also provided adequate information on how soil field capacity below 70% affects choy sum productivity. Future work is being planned to model for water stress in choy sum grown in the open field conditions using the different parameters measured in this study.

## Figures and Tables

**Table 1:** Climatological information collected at various phases of crop development.

Growth Stages	RH (%)	Ra (w/m^2^)	Ta (°C)	VPD (kpa)
Initial growth stage	62.3	300.31	25.4	0.33
Vegetative stage	67.6	297.98	23.4	0.39
Flowering stage	57.1	276.1	22.7	0.37

All values are the mean of each parameter. RH is the relative humidity; Ra is the solar radiance; Ta is the air temperature; VPD is the vapor pressure deficit.

**Table 2:** Soil physicochemical parameters of the experimental site at a depth of (0–30 cm).

Soil Properties	Values
Soil Texture	Sandy loam soil
pH	5.54 ± 0.11
Organic Matter (g/kg)	15.8 ± 1.0
Total N (g/kg)	1.26 ± 0.02
Total P(g/kg)	0.88 ± 0.21
Total K(g/kg)	9.48 ± 0.03
Alkalized N(mg/kg)	451 ± 1.8
Available P (mg/kg)	185 ± 2.53
Available K (mg/kg)	439 ± 43.4
Sand (%)	47.6 ± 0.06
Clay (%)	17.3 ± 1.54
Silt (%)	35.1 ± 0.3
Bulk density (g/cm^3^)	1.34 ± 0.21
Field capacity (%)	0.21 ± 0.03
Wilting point (%)	0.09 ± 0.02
Saturation point (%)	0.49 ± 0.14

Note: all values above are the means of three replicates for each property.

**Table 3:** Formulae and references for the spectral vegetative indices utilized in the study.

Index	Formulae	References
Normalized difference vegetation index (NDVI)	$\mathrm{NDVI}=\frac{\;(R_{800}-R_{670})}{\;\left(R_{800}+R_{670}\right)}$	[34]
Renormalized difference vegetative index (RDVI)	$\mathrm{RDVI}=\frac{\left(R_{800}-R_{670}\right)}{{\left(R_{800}+R_{670}\right)}^{0.5}}$	[35]
Green Chlorophyll index (CL_green_)	${\mathrm{CL}}_{\mathrm{green}}=\frac{\left(R_{750}\right)}{\left(R_{550}\right)}-1$	[36]
Normalized difference water index (NDWI)	$\mathrm{NDWI}=\frac{\;(R_{800}-R_{1240})}{\;\left(R_{800}+R_{1240}\right)}$	[37]
Normalized photochemical reflective index (PRI_norm_)	${\mathrm{PRI}}_{\mathrm{norm}}=\frac{\mathrm{PRI}}{\left(\mathrm{RDVI}*\frac{R_{700}}{R_{670}}\right)}$	[38]
Photochemical reflective index (PRI)	$\mathrm{PRI}=\frac{\;(R_{531}-R_{570})}{\;\left(R_{531}+R_{570}\right)}$	[35]

*R* represents reflectance.

**Table 4:** Correlation between CWSI and Vis.

Vegetative Indices	CWSI_W_
PRInorm	0.71 ***
NDVI	0.80 ***
NDWI	0.87 ***
CL_green_	0.62 ***

*** *p* ≤ 0.001, data used for correlation are mean values during the plant growth, each treatment represents 7 data points giving a total of 28 data points for all four main treatments.

**Table 5:** The ANOVA analysis on the main effects of different irrigation regimes on the choy sum roots’ morphology.

Treatments	ARL (cm)	RLD (g/cm^3^)	RWD (mg/cm^3^)	RD (mm)	TSA (m^2^)	RFW (g)	RDW (g)	SRL (m/g)
60–70% FC (SWS)	3921 ± 32.1 ^d^	1.38 ± 0.72 ^c^	0.06 ± 0.02 ^d^	0.14 ± 0.04 ^d^	0.03 ± 0.91 ^c^	1.67 ± 0.16 ^c^	0.17 ± 0.08 ^d^	273.09 ± 31.34 ^c^
70–80% FC (MWS)	6505 ± 57.3 ^c^	2.28 ± 0.34 ^b^	0.08 ± 0.06 ^c^	0.22 ± 0.02 ^c^	0.06 ± 0.23 ^b^	2.35 ± 0.49 ^b^	0.23 ± 0.03 ^c^	285.26 ± 42.33 ^b^
80–90% FC (LWS)	6830 ± 73.6 ^b^	2.40 ± 0.43 ^b^	0.09 ± 0.05 ^b^	0.24 ± 0.01 ^b^	0.06 ± 1.43 ^b^	2.27 ± 0.25 ^b^	0.26 ± 0.01 ^b^	265.71 ± 48.26 ^d^
90–100% FC (NWS)	7870 ± 96.8 ^a^	4.75 ± 0.97 ^a^	0.10 ± 0.03 ^a^	0.31 ± 0.07 ^a^	0.08 ± 3.95 ^a^	2.42 ± 0.63 ^a^	0.28 ± 0.02 ^a^	334.91 ± 67.43 ^a^

Different letters within a column refer to significant differences (*p* < 0.05) among treatments: ARL Average Root Length; RLD—Root length density; RWD—Root weight Density; RD—Root Diameter; TSA—Total Surface Area; RFW—Root Fresh Weight; RDW—Root Dry Weight; SRL—Specific Root Length Note. Each value is the mean data collected on four plants from each treatment.
